# Reframing the relationship between the economy and health

**DOI:** 10.2471/BLT.24.291710

**Published:** 2024-05-01

**Authors:** Ritu Sadana, Serife Genc Illeri, Maksym Obrizan, Alberto Huitron, Devika Dutt, Roberto Duran-Fernandez

**Affiliations:** a Secretariat, WHO Council on the Economics of Health for All, World Health Organization, Avenue Appia 20, 1211 Geneva 27, Switzerland.; b Department of Economics, Istanbul Technical University, Istanbul, Türkiye.; c Kyiv School of Economics, Kyiv, Ukraine.; d Washington, DC, United States of America.; e Department of International Development, Kings College London, London, England.; f Tecnológico de Monterrey, Escuela de Gobierno y Transformación Pública, Monterrey, Mexico.

The World Health Organization (WHO) Council on the Economics of Health for All asserts that investing for health is key to creating an inclusive economy and that, if health for all is the overall goal, the economy must be restructured to serve it.^1^ This need for developing alternatives calls for a critical examination of the relationship between the economy and health, underlying assumptions and available policy levers and tools.

The council argues that the experience of the coronavirus disease 2019 (COVID-19) pandemic reflected the world’s collective failure to govern economies for the common good,^2^ and laid bare the profound consequences of ignoring the interrelationship between economies and health. As a response, the council’s brief *Valuing health for all: rethinking and building a whole-of-society approach* proposes three value sets to align goals that promote the health of people and of the planet.^3^ The first set is valuing planetary health, including essential common goods such as clean water, clean air and a stable climate, with respect to planetary and local ecological boundaries – all considered vital determinants of health. The second set is valuing the diverse social foundations and activities that promote equity, including social cohesion, supporting people in need and enabling communities to thrive, reflecting the theory of change underpinning action on the social determinants of health. The third is valuing human health and well-being from a rights-based foundation, with the goals that governments commit to the right to health, that every person can prosper physically, mentally and emotionally, and that all people are endowed with the capabilities and freedom needed to lead lives of dignity, with opportunity and within a community.

The council’s framework^3^ builds on these values, as well as theory and arguments advanced by those who have studied the relationship between the economy and health from a more integrated perspective. Studies include the Commission on Macroeconomics and Health that focused on new mechanisms to finance essential health services for people who are economically disadvantaged, particularly in low- and middle-income countries;^4^ the Commission on Social Determinants of Health that promoted a whole-of-society approach to lift up the entire social gradient to build just and fair societies, relevant to all countries;^5^ and the Commission on the Measurement of Economic Performance and Social Progress that consolidated evidence that measures beyond gross domestic product must be the basis for policies and actions to advance human development.^6^

The WHO Council on the Economics of Health for All provides leadership^3^^,^^7^^–^^9^ and bold recommendations,^10^ highlighting that finance and economic levers can be shaped towards health for all, aligning economic, social, environmental and health goals whether at global, national or local levels. For example, national budget processes can be designed to focus on well-being outcomes that have an impact on people’s lives and move away from silos that still exist between and within sectors.^11^ Moreover, policy-makers can establish and choose to increase domestic and cross-border investments in health, including in low-resource settings. However, these are policy choices that must be continuously advocated for and implemented through collaborative mechanisms that also engage people and build trust.

Strategic reforms in finance, legal and economic sectors across public, private and nongovernmental sectors, can shape and inform cogent policy-making, with the goal of improving the lived experiences and health and well-being of communities, households and individuals.^12^ This approach in practice means broadening the tax base; introducing taxation that is more progressive; increasing the population’s financial literacy; enhancing financial inclusion; strengthening public sector capacity in building equitable financial architectures; and eliminating financial obstacles in relation to access to health services. These actions can be operationalized through multiple policy decisions, such as those of central banks and in the design of pension and other social protection systems.

The council confirms that positive change is possible, but that it requires urgent, systematic action. Policy-makers need to break down problems into their root causes, identify entry points for change, search for possible solutions, and act and reflect upon the learnings from local to global contexts, using participatory approaches. Further refinement of the framework will help build an economy for health for all.
